# *In vitro* studies on curcumin-chitosan-Multiwalled Carbon Nanotubes (MWCNT) cytotoxicity against A549 (lung cancer cell line) HCT 116 (colorectal cancer cell line) and PANC1 (pancreatic cancer cell line)

**DOI:** 10.1016/j.toxrep.2026.102254

**Published:** 2026-04-15

**Authors:** Manar M. Rabba’a, Rund A. Abu-Zurayk, Yaser K. Bustanji, Bashaer Abu-Irmaileh, Saida Abu Mallouh, Aya Khalaf

**Affiliations:** a Biology Department, School of Science, The University of Jordan, Amman, Jordan; b Faculty of Pharmacy, Department of Basic Pharmaceutical Sciences, Isra University, Amman, Jordan; c Hamdi Mango Center for Scientific Research, The University of Jordan, Amman, Jordan; d Research Institute of Medical and Health Sciences, University of Sharjah, Sharjah, United Arab Emirates; e College of Medicine, University of Sharjah, Sharjah, United Arab Emirates; f School of Pharmacy, The University of Jordan, Amman, Jordan; g Allied Sciences Department, Faculty of Arts and Sciences, Al-Ahliyya Amman University, Amman 19328, Jordan

**Keywords:** Nanocomposites, Biocompatibility, Curcumin, MWCNT, Drug delivery, Cancer therapy

## Abstract

The use of nanoparticles in cancer research has garnered significant interest due to their exceptional physicochemical properties. Among these, carbon nanotubes have shown potential in biomedical applications; however, their hydrophobic nature limits dispersion in aqueous environments. Therefore, surface functionalization is important to improve the dispersibility and biocompatibility of Multiwalled Carbon Nanotubes (MWCNTs). This study aimed to non-covalently functionalize MWCNTs with chitosan, characterize the resulting nanocomposites, and evaluate the *in vitro* cytotoxicity of oxidized MWCNTs, curcumin-loaded MWCNTs, and chitosan-functionalized MWCNTs with or without curcumin against lung, pancreatic, and colorectal cancer cell lines. The prepared samples were characterized for crystallinity, particle size, and surface charge using X-ray Diffraction (XRD) and Dynamic Light Scattering (DLS). The resulting curcumin-chitosan-MWCNT formulation demonstrated an entrapment efficiency of 99.1%, a particle size of 850 nm, and a surface area of 52.73 m²/g. The IC₅₀ of curcumin-chitosan-MWCNT was 67 μg/mL for PANC-1 cells, compared to 227.6 μg/mL for fibroblasts, 71.4 μg/mL for HCT116, and 148.6 μg/mL for A549 cells. In conclusion, the combination of curcumin, chitosan, and MWCNT significantly reduced cancer cell viability and demonstrated selective in vitro cytotoxicity, particularly against PANC-1 cells. These findings suggest that the developed formulation may warrant further investigation as a potential nanocomposite platform for anticancer applications**.**

## Highlights

• MWCNTs were non-covalently functionalized with chitosan to improve biocompatibility.
• Curcumin was efficiently loaded onto chitosan–MWCNT with an entrapment efficiency of 99.1%.
• The nanocomposite exhibited a size of 850 nm and a surface area of 52.73 m²/g.
• Curcumin–chitosan–MWCNT showed potent cytotoxicity against A549, HCT116, and PANC-1 cells.
• Enhanced anticancer activity indicates potential for pancreatic cancer drug delivery.

## Graphical Abstract

**Figure:**
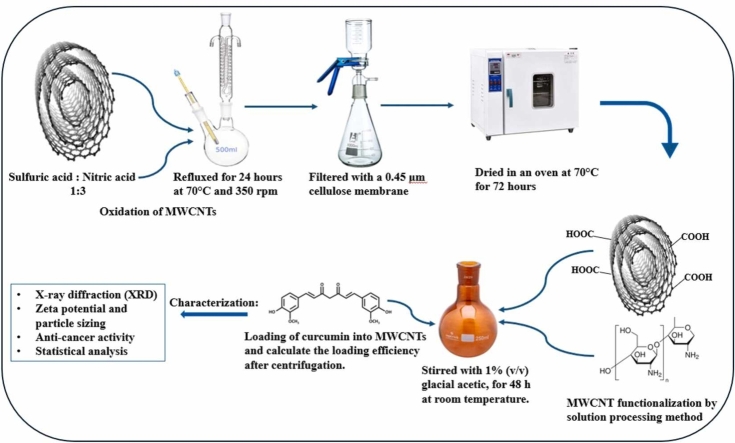

## 1 Introduction

Lung, colorectal, and pancreatic cancers are leading global causes of death, posing significant public health challenges [1]. Despite advances in therapy, many patients still face poor diagnoses [2], highlighting the need for new strategies [3]. Recent efforts focus on nanotechnology and natural compounds [4], particularly curcumin, in cancer treatment [5].

Nanotechnology has advanced multiple fields [6], including medicine. Nano- sized materials exhibit unique properties [7], enabling design of specific materials [8]. Carbon nanotubes (CNTs) are cylindrical carbon structures with exceptional electrical, mechanical, and thermal properties, making them promising nanomaterials in research and medicine [9]. CNTs enhance drug delivery efficiency compared to conventional approaches due to their targeted drug release and high loading capacity [10]. Multi- Walled Carbon Nanotubes (MWCNTs) are notable for their unique hollow structure and high surface area, aiding drug delivery and cancer therapy. While MWCNTs are generally non- toxic [11], prolonged exposure at high concentrations can cause adverse effects, such as genotoxicity and oxidative stress [12]. Various moieties, including curcumin, can be attached to MWCNTs to mitigate these effects [5]. Curcumin, a lipophilic polyphenol from turmeric, has diverse pharmacological benefits but limited clinical use due to low bioavailability and rapid metabolism [5]. Its hydrophobic nature restricts cellular uptake, necessitating structural modifications for improved pharmacological properties and selective cancer targeting [13]. While curcumin' s anticancer effects on normal cells remain debated, research shows varying cytotoxic effects on fibroblasts based on concentration [14].

The optimal drug delivery system should focus on targeted release to reduce toxicity. CNT functionalization can enhance drug distribution and cell penetration [11]. Chitosan, a biodegradable polymer, is commonly used to enhance stability and bioavailability of drugs [15]. Functionalizing curcumin with chitosan can yield more effective therapeutic applications [13]. Curcumin targets multiple pathways involved in cancer progression [16]. This study investigates the cytotoxic effects of a formulation combining curcumin, chitosan, and MWCNTs against pancreatic (PANC 1), lung, and colorectal cancer cell lines. *In vitro* studies will elucidate the formulation' s mechanisms and potential as a novel treatment for these aggressive cancers, advancing targeted therapies.

## 2 Materials

The below-listed materials were utilized without any additional modifications unless otherwise stated.

Multi-walled carbon nanotubes with (95% purity, 30–50 nm outside diameter and 10–20 μm length) from US Research Nanomaterials, Inc. USA. Nitric acid (70 wt%) from (CARLO ERBA, France). Sulfuric acid (98 wt%), Anhydrous N, N-Dimethylformamide (DMF) 99.8%, oxalyl chloride ≥ 99%, and curcumin powder (Cat # C1386) were purchased from (Sigma Aldrich, Beijing, China). Low molecular weight chitosan (Genochem World, Batch # 412QEG). Dulbecco’s phosphate-buffered saline (pH 7.4, Euro clone, Cat no. ECB4004L). Dimethyl sulfoxide (DMSO) cell culture reagent was obtained from (Santa Cruz Biotechnology, USA). Cellulose membrane filters (0.45 μm pore diameter), glacial acetic acid, NaOH, nitrogen gas, absolute ethanol, the dye solution (MTT) was obtained from (Promega, USA). [3- (4,5-dimethylthiazol-2-yl) 2,5-diphenyltetrazolium bromide] kit, fibroblast cell line, PANC-1 pancreatic cancer cell line, A549 lung cancer cell line, and HCT116 colorectal cancer cell line, Iscove’s media, Gibco™ Roswell Park Memorial Institute (RPMI) 1640 Medium and Gibco™ Dulbecco's Modified Eagle Medium (DMEM) were obtained from (Thermo Fisher Scientific, USA), MEM nonessential amino acids 100X obtained from Euro Clone S.p.A. Italy, trypsin obtained from Biowest the serum specialist, Penicillin-Streptomycin solution 100X obtained from Euro Clone S.p.A. Italy. and fetal bovine serum (FBS standard) obtained from PAN™ Biotech.

## 3 Methods

### 3.1 Samples preparation

All samples were performed according to our previously published protocol [4].

**Oxidation of pristine multi-walled carbon nanotubes** (MWCNTs): MWCNTs were oxidized by dispersion of 0.2 g of MWCNTs in a 100 mL acid mixture [H₂SO₄/HNO₃ (1:3), v/v)] under reflux for 24 h at [temperature, 70 °C, and 350 rpm]. The dispersion was then diluted for 1000 mL with distilled water, filtered through a 0.45 μm cellulose membrane, and repeatedly washed by deionized water to have acid-free MWCNTs until neutral pH was achieved. Finally, the MWCNTs were exfoliated from the membrane and dried in an oven at 70 ℃ for 72 h.

**Functionalization of MWCNT** by chitosan to obtain Chitosan-MWCNT composite was carried out by either the solution-processing method or chemical functionalization. In **solution processing method** [Five mg/mL of low molecular weight chitosan was dissolved in 1% (v/v) glacial acetic acid, mixed with 2.5 g/mL of oxidized MWCNTs, sonicated for 20 min, and stirred for 48 h. at room temperature, then 1% (w/v) NaOH was used to collect chitosan bounded with MWCNTs (chitosan-MWCNT) washed several times with deionized water and centrifuged at 4000 rpm and 4 ℃ to remove any unbounded chitosan, then allowed to dry at room temperature]. While in **Chemical Functionalization** A mixture of 120 mg of previously prepared oxidized MWCNT and 80 mL of DMF was sonicated for 20 min, then 1 mL oxalyl chloride was added dropwise to the suspension under nitrogen gas. This mixture was stirred at 350 rpm in an ice bath for 2 h. then it was stirred at room temperature for another 2 hr, followed by overnight stirring at 70 ℃ to remove excess oxalyl chloride. After that, a mixture composed of 490 mg of low molecular weight chitosan and 40 mL of DMF was sonicated for 20 min then added to the previous mixture (MWCNT, DMF, and Oxalyl chloride) and stirred for 48 h at 110 ℃. The resulting mixture was then filtrated and washed until no chitosan residues remained in the filtrate, finally, the particles were vacuum dried for 4 hr.

**Loading of curcumin into MWCNTs:** 40 mg of oxidized MWCNT, chitosan-MWCNT, and chitosan-MWCNT-oxalyl-Cl each were solely dispersed in 20 mL of absolute ethanol, sonicated for 30 min. This step was repeated for two different batches each, as it was applied in dark and light conditions. Forty milligrams of curcumin powder were weighed in 6 tubes, each one was dispersed in 20 mL of absolute ethanol and sonicated for 30 min. Then the two solutions (curcumin and MWCNT) were mixed, sonicated for 30 min, and stirred at 350 rpm for a 16 h on either dark or light to load the curcumin on the surface of MWCNTs as presented in Table 1. Each solution was centrifuged at 4℃ and 3000 rpm for 10 min and the supernatant was used to measure the loading efficiency with the aid of a spectrophotometer at 428 nm, whereas the resulted combinations of curcumin-MWCNT (dark), curcumin-MWCNT (light), curcumin-chitosan-MWCNT (dark), curcumin-chitosan-MWCNT (light), curcumin-chitosan-MWCNToxalyl-Cl (dark) and curcumin-chitosan-MWCNT-oxalyl-Cl (light) samples were washed with deionized water and dried at room temperature for further use.

**Table 1:** Loading conditions of curcumin onto Oxidized- MWCNTs and chitosan-MWCNTs under light and dark conditions. Curcumin was loaded onto oxidized MWCNTs, chitosan–MWCNTs prepared by solution processing, and chitosan–MWCNTs prepared via oxalyl chloride. Light and dark conditions indicate drug-loading procedures performed in the presence or absence of light to evaluate the influence of curcumin photosensitivity on loading behavior.

Drug combination		Loading condition
Curcumin	Oxidized-MWCNT	Light
		Dark
Curcumin	Chitosan-MWCNT (Solution processing)	Light
		Dark
Curcumin	Curcumin Chitosan-MWCNT (oxalyl-Cl)	Light
		Dark

#### 3.1.1 Determination of curcumin loading efficiency onto MWCNT

The supernatant resulted from the previous step was diluted to 1:1000 and the absorbance of the diluted sample was measured at 428 nm, using a spectrophotometer, then the absorbance mean of the diluted supernatant was applied to the equation resulted from the curcumin calibration curve, after that the resulted value was multiplied by the dilution factor (1000) and subtracted from the final concentration of curcumin used in loading experiment. The loading efficiency was calculated according to Eq. (1) [15].

(1) Loading efficiency = (C_t_-C_s_)/C_t_ x 100%

Where Ct is the total amount of curcumin used, and Cs is the amount of curcumin in the supernatant.

#### 3.1.2 Determination of curcumin Entrapment efficiency onto MWCNT

To measure the entrapment efficiency of curcumin on MWCNTs ethanolic dispersions, a concentration of 1 mg/mL of each prepared sample previously listed in Table 1. After that, each dispersion was sonicated for 20 min, then centrifuged at 4 ℃ and 4000 rpm for 30 min. The absorbance of curcumin in the supernatant (free drug) was measured by spectrophotometer at 428 nm. Then the entrapment efficiency of curcumin in each drug was measured using Eq. 2. [15].

(2) *Entrapment efficiency = (WC*_*i*_*-WC*_*f*_*)/WC*_*i*_*x 100%*

Where WC_i_ is the initial weight of curcumin, and WC_f_ is the weight of free curcumin.

### 3.2 Characterization

#### 3.2.1 X-ray diffraction (XRD)

Patterns of pristine MWCNTs, oxidized MWCNT, chitosan, curcumin, chitosan-MWCNT, curcumin-chitosan-MWCNT, and curcumin-MWCNT were analyzed across a 2 Ɵ scanning range with a 0.02 step size using a Shimadzu X-ray spectrophotometer at ʎ= 1.4 Å [17]. This method characterized the samples' structural properties.

#### 3.2.2 Zeta potential and particle sizing

Particle size and zeta potential were examined to assess the impact of MWCNT chemical treatments on surface charge and size. Hydrodynamic radius measurements of diluted samples (1:20) were taken using a Malvern Zetasizer Nano with a 633 nm He-Ne laser for evaluating hydrodynamic diameter and sample stability at 25 °C. This method was used to qualitatively analyze how surface charge affects dispersion quality [18].

#### 3.2.3 Anti-cancer activity

##### 3.2.3.1 Cytotoxicity test

The effects of oxidized MWCNT, chitosan, curcumin, chitosan-MWCNT, curcumin-chitosan-MWCNT, and curcumin-MWCNT on the viability of Human Dermal fibroblast cells (**PCS-201–012** ™), PANC-1 (**CRL-1469** ™ pancreatic cancer cell line), A549 (CCL-185 ™lung cancer cell line), and HCT116 (CCL-247EMT colorectal cancer cell line) were evaluated using viability/cytotoxicity (MTT colorimetric assay) kit that analyzes the integrity of mitochondrial function by producing formazan crystals, which are proportionally related to the cell viability as the higher crystal production, exhibit higher cell viability.

Each cell line was cultured in complete media: fibroblast in Iscove’s media, PANC-1, A549 [19], and HCT116 in DMEM-high glucose at 37°C with 5% CO₂ [20]. When 80% confluent, cells were trypsinized and counted; 8000 cells/well (well:100 µL) were seeded in 96-well plates and incubated for 24 h for adherence. All stocks were in DMSO at 20 mg/mL. Cells were treated with 100 µL of serial dilutions of oxidized-MWCNT, chitosan, curcumin, chitosan-MWCNT, curcumin-chitosan-MWCNT, and curcumin-MWCNTs starting from 400 μg/mL down to 12.5 μg/mL, totaling 200 µL per well, with 1% DMSO. Control wells received 1% DMSO only. Plates were incubated for 72 h; IC50 was recorded using Cell Titer 96® Non-Radioactive Assay according to the manufacturer’s instructions, and readings were taken at 570 nm with an ELISA microplate reader.

#### 3.2.4 Note

For all experiments, equal-mass controls were included to allow direct comparison between free components and composite formulations. Free curcumin, free chitosan, and oxidized MWCNTs were tested at concentrations corresponding to their respective mass fractions within the curcumin–MWCNT and curcumin–chitosan–MWCNT nanocomposites at each dose level. Untreated cells served as the negative control and were assigned 100% viability. All viability values were normalized to the untreated control to ensure consistency across experimental groups and cell lines.

Also the concentration range used in this study (12.5–400 µg/mL) was established based on preliminary dose-finding experiments conducted to define the dynamic response window of the tested formulations. These pilot studies enabled the identification of concentrations spanning minimal to significant cytotoxic effects, thereby ensuring robust dose–response modeling and accurate IC₅₀ determination across different cell lines.

#### 3.2.5 Statistical analysis

Each treatment was replicated three times; statistical analysis of the data and the IC_50_ was calculated using GraphPad prism software with p value of (p < 0.05). The results were expressed as Mean ± SEM (Standard Error of the Mean).

## 4 Results and discussion

### 4.1 Oxidation, Functionalization, Loading and Entrapment Efficiency of curcumin on MWCNTs

Oxidation of MWCNTs through acid refluxing is expected to remove impurities and produce MWCNT-COOH [21]. These functionalized MWCNTs are suitable for targeted drug delivery due to their biocompatibility [21], [22], [23], [24], [25].

Encapsulating MWCNTs with chitosan, a biodegradable polysaccharide, leads to MWCNT nanocomposites for anticancer drug delivery, verified by Zeta potential and XRD results [22]. Chitosan's biomedical properties arise from its β-(1–4)-linked D-glucosamine structure, offering non-toxicity and compatibility [23].

Encapsulation has been widely investigated as a strategy to improve the stability and controlled release of bioactive molecules [11]. Previous studies have suggested that chitosan’s protonated amino groups may promote favorable interactions with negatively charged biomolecules and biological interfaces [23], [24]. In the present study, MWCNTs were oxidized and non-covalently coated with chitosan, which improved hydrophilicity and reduced aggregation, both of which may influence dispersion behavior and nano–bio interactions. Chitosan is also known to improve colloidal stability of MWCNT-based systems and is eventually metabolized to N-acetyl glucosamine [25]. However, cellular uptake and intracellular delivery were not directly assessed in the current work; therefore, no conclusions regarding uptake enhancement can be drawn from these data.

As shown in Table 1, the highest curcumin loading efficiency was 54% for oxidized-MWCNTs and 99.1% for chitosan-MWCNTs, whereas steric hindrance may limit curcumin loading on oxidized MWCNTs [26]. Severe acid oxidation can disintegrate CNTs [27], impairing drug delivery effectiveness [28]; however, their high aspect ratio allows significant payload capacity and efficient cell penetration [29].

The high entrapment efficiency observed (Table 2) likely arises from curcumin's full exposure to the MWCNT surface in ethanol [30]. Its structure, with two benzene rings and an ethylenic linkage, allows easy adsorption onto MWCNT under ultrasonic energy through hydrogen bonds between the carboxyl groups of the functionalized MWCNTs and the phenolic hydroxyl group of curcumin [31]. In addition, van der Waals interactions between hydrophobic curcumin and MWCNT enhance entrapment [32]. Curcumin possible loading into MWCNT cavities, contribute to effective encapsulation [9], [26], [27].

**Table 2:** Loading efficiency and entrapment efficiency of curcumin on oxidized multiwalled carbon nanotubes (MWCNTs) and chitosan-functionalized MWCNTs prepared by solution processing or oxalyl chloride activation under light and dark conditions. Data are presented as mean ± SEM (standard error of the mean) (n = 3). Light and dark conditions refer to drug-loading procedures performed in the presence or absence of light to evaluate the influence of curcumin photosensitivity on loading and entrapment behavior.

Drug Combination		Loading Condition	Loading Efficiency %	Entrapment Efficiency %
Curcumin	MWCNT	Light	54 ± 0.79	80.9 ± 0.0021
		Dark	48.5 ± 0.53	93.6 ± 0.0021
Curcumin	Chitosan-MWCNT (Solution-processing)	Light	33.3 ± 0.23	98 ± 0.014
		Dark	27.3 ± 0.47	96.7 ± 0.0021
Curcumin	Chitosan-MWCNT (Oxalyl-Cl)	Light	34.7 ± 0.68	99.1 ± 0.001
		Dark	38.7 ± 0.41	97.4 ± 0.006

**Table 3:** Hydrodynamic size (nm), polydispersity index (PdI), and zeta (ζ) potential of pristine and oxidized multiwalled carbon nanotubes (MWCNTs), chitosan, curcumin, and their corresponding nanocomposites. Particle size and PdI were determined by dynamic light scattering (DLS), and ζ-potential values were measured by electrophoretic light scattering (n = 3). Measurements were performed to evaluate colloidal stability and surface charge characteristics of the different formulations.

Code	Name	Size ± PdI	ζ potential
1	Pristine –MWCNT	588.3 ± 0.64	-20.1
2	Oxidized- MWCNT	351.5 ± 0.53	-14.6
3	Chitosan	5758 ± 0.46	+ 1.8
4	Curcumin	715.4 ± 0.32	-15.2
5	Chitosan-MWCNT	764.5 ± 0.36	-6.0
6	Curcumin-MWCNT	285.2 ± 0.34	-21.4
7	Curcumin-chitosan-MWCNT	850 ± 0.55	-6.9

### 4.2 Characterization

#### 4.2.1 X-Ray diffraction analysis (XRD)

**Fig. 1:**
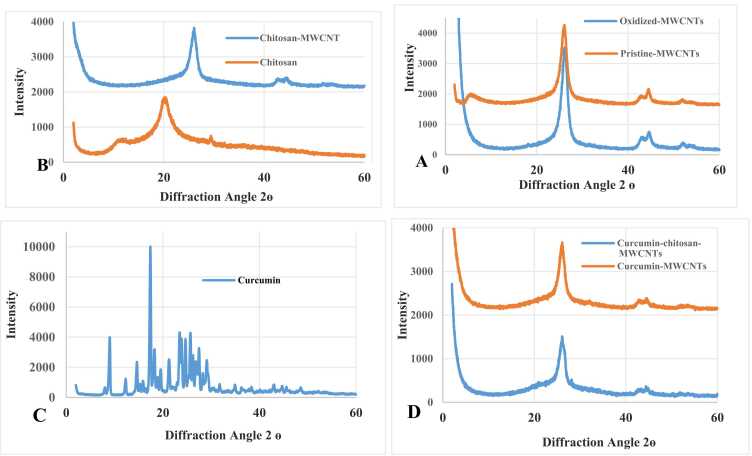
X-ray diffraction (XRD) patterns of (A) pristine MWCNTs and oxidized MWCNTs. (B) chitosan and chitosan-functionalized MWCNTs. (C) curcumin (D) curcumin- MWCNTs and curcumin- chitosan- MWCNTs. The diffraction patterns were recorded as intensity versus diffraction angle **(2θ).** The broadening and reduced intensity of characteristic crystalline peaks in the nanocomposites indicate successful surface functionalization and the formation of an amorphous or partially amorphous complex, confirming effective curcumin encapsulation and interaction with chitosan-MWCNTs.

XRD analysis reveals the crystal structure and crystallinity of carbon nanotube materials through intensity patterns of diffracted X-rays versus diffraction angle (2ϴ) from 0◦ to 60• (Fig. 1A-1D). Pristine-MWCNT, oxidized-MWCNT, and chitosan-MWCNT display sharp peaks at 2θ values of 26.24º, while curcumin-MWCNT and curcumin-chitosan-MWCNT appear at 26.08º and 26.16º, respectively. These changes indicate surface activation without compromising CNT alignment [33], preserving the intensity indicative of CNT crystalline nature [11]. Low molecular weight chitosan shows a broad peak at 2θ value of 20.58º, indicating low crystallinity and amorphous state [34]. Connecting chitosan with MWCNT induces a structural phase change, as chitosan's -OH groups interact with CNT functional groups, leading to new low-intensity peaks and confirming reduced crystallinity of chitosan-MWCNT compared to oxidized MWCNT, verifying functionalization. Curcumin's XRD pattern displays intense peaks at 2ϴ values of 9.02º, 17.4º, 23.54º, 25.7º, and 27.46º, confirming it as an organic crystalline material. These peaks fade with oxidized-MWCNT or chitosan-MWCNT, likely due to insufficient curcumin for detection or loss of crystalline structure during synthesis [34]. Curcumin-chitosan-MWCNT and curcumin-MWCNT patterns reveal new low-intensity peaks, signaling reduced crystallinity in chitosan-MWCNT due to grafted chitosan, confirming an amorphous complex in the nanocomposites [11].

#### 4.2.2 Particle size and zeta potential

Size and zeta potential results are in Table 2, with size reported as (value ± PdI), where PdI is Polydispersity Index. A PdI> 0.7 indicates a broad size distribution. The size reduction for oxidized- MWCNTs (351.5 nm) likely results from impurity removal and strong acid used during oxidation [35]. Functionalizing oxidized-MWCNT with chitosan increased average sizes to 764.5 nm, curcumin- MWCNTs to 285.2 nm, and curcumin-chitosan- MWCNTs to 850 nm due to curcumin encapsulation [36]. Significant size differences were noted among curcumin-chitosan-MWCNTs, oxidized-MWCNTs, chitosan-MWCNTs, and curcumin-MWCNTs [37] due to the addition of chitosan. Penetration of a hydrophobic modified curcumin and chitosan nanoparticles through the cell membrane by endocytosis is influenced by a variety of factors, the most important of which are their size, shape, and cell line characteristics [38]. Regarding the particle size dependence on endocytosis is linked with cell membrane ability to wrap around different sized particles, Optimal size for cell internalization is 380 nm to 780 nm, correlating with vascular cut-off in tumors [39]. While, Rejman et al. [40] reported that large particles with size up to 1 μm were taken up by macropinocytosis and phagocytosis [41], [42].

DLS size values indicate changes in MWCNT diameter from chemical treatments and assembly [41]. Zeta potential distributions for pristine-MWCNT, oxidized-MWCNT, chitosan, curcumin, and their derivatives were −20.1, −14.6, + 1.8, −15.2, −6, −6.9, and −21.4 (Table 2). The narrow peaks indicate mono-dispersion on surface charge [42]. Nanoparticle stability in colloidal systems is influenced by zeta potential [43], with higher charge magnitudes increasing electrostatic repulsion and reducing aggregation [44]. Zeta potential reflects the electric charge strength between particles, indicating stability. A good zeta potential value is far from zero, which is consistent with our results. Approaching zero could lead to aggregation and unstable nanosuspensions. Therefore, a larger charge enhances particle stability through increased resistance among particles [45].

To contextualize the physicochemical characteristics of the developed nanocomposite, the obtained particle size and ζ-potential values were compared with those reported for similar curcumin-based nanocarrier systems.

The Curcumin–MWCNT formulation exhibited a particle size of approximately 285 nm, which falls within the nanoscale range commonly reported for curcumin-based nanocarrier systems (100−300)nm [46]. Previous studies have associated this size range with favorable physicochemical behavior; however, particle size alone cannot be used to infer cellular internalization or delivery performance in the present study. For instance, curcumin-loaded chitosan nanoparticles with a mean size of approximately 200–300 nm have been reported to exhibit improved stability and enhanced anticancer activity compared to free curcumin [47].

In contrast, the Curcumin–chitosan–MWCNT nanocomposite in the present study demonstrated a larger particle size (∼850 nm), which may be attributed to chitosan coating and partial aggregation. Similar increases in particle size following polymer functionalization have been reported in chitosan-based nanocarrier systems, where coating and crosslinking processes contribute to particle enlargement and increased polydispersity [48]. Despite this increase, polymer-coated nanocarriers may exhibit altered surface properties and dispersion behavior due to enhanced surface functionality; however, the biological implications of these changes were not directly evaluated in this study.

The observed shift in ζ-potential from −21.4 mV (Curcumin–MWCNT) to −6.9 mV (Curcumin–chitosan–MWCNT) is consistent with previous findings demonstrating that chitosan incorporation reduces the magnitude of negative surface charge due to electrostatic interactions between the cationic polymer and negatively charged nanostructures. Also Such modulation of surface charge has been associated in previous reports with altered colloidal stability and nano–bio interface behavior [49]; however, these effects were not directly linked to cellular uptake or delivery efficiency in the present work.

Overall, previously reported curcumin nanocarriers—including polymeric nanoparticles, lipid-based systems, and hybrid nanocomposites—typically exhibit particle sizes in the range of 100–300 nm and ζ-potential values between −30 mV and + 30 mV, depending on formulation composition and surface modification [46]. Therefore, while the Curcumin–MWCNT formulation aligns closely with the optimal nanoscale range, the Curcumin–chitosan–MWCNT system represents a formulation trade-off, where increased particle size is accompanied by altered surface properties that may influence biological interactions. It is important to note that direct comparison across studies remains inherently limited due to variations in preparation methods, experimental conditions, and measurement techniques. Accordingly, these comparisons are intended to provide a general contextual framework rather than a strict performance ranking.

#### 4.2.3 Cytotoxicity

The MTT assay evaluated the cytotoxicity of oxidized-MWCNTs, chitosan-MWCNTs (with and without curcumin), curcumin-MWCNTs, and free chitosan, and curcumin. To prevent false positives in MTT assays from formazan sticking to CNTs, cells were rinsed with phosphate-buffered saline prior to MTT addition [50].

**Fig. 2:**
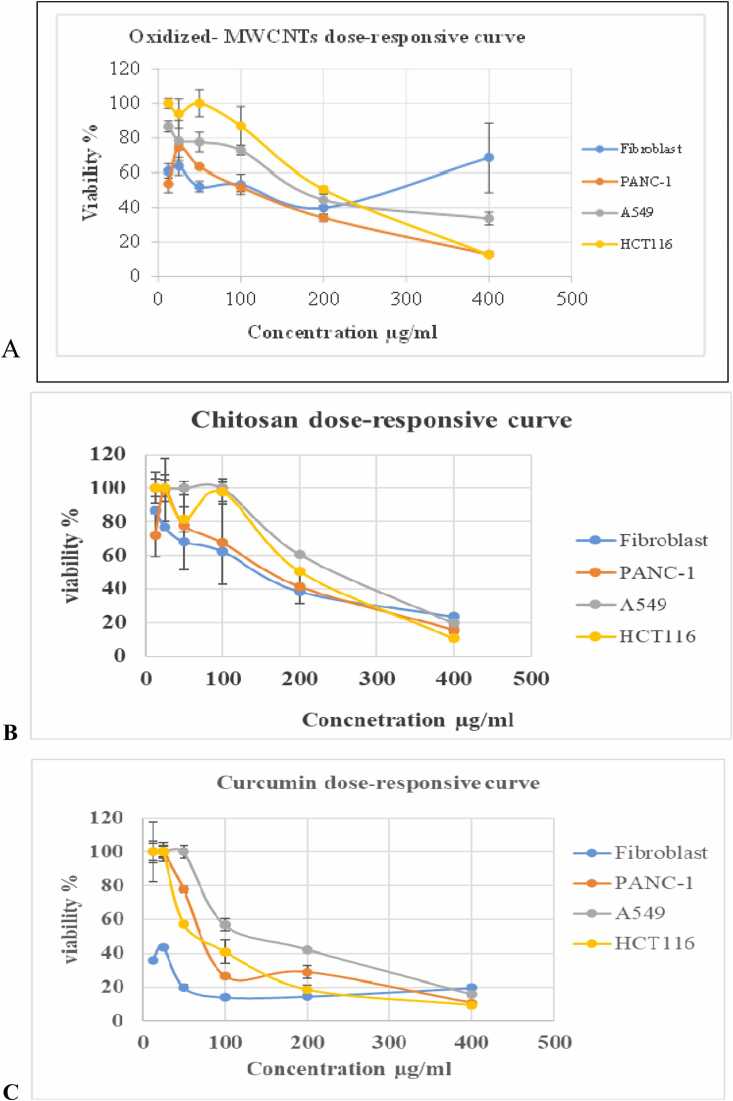

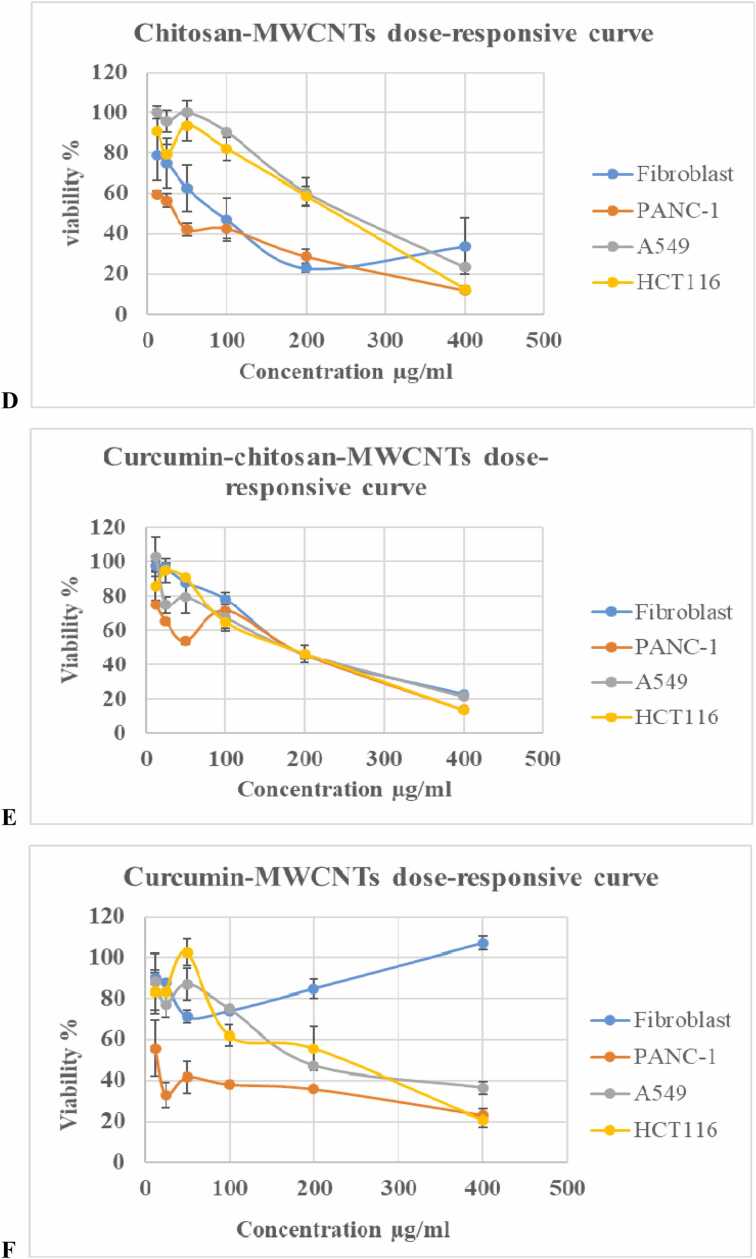
Dose–responsive curves of cell viability after treatment with different formulations. (A) Oxidized MWCNTs, (B) chitosan, (C) curcumin, (D) chitosan–MWCNTs, (E) curcumin–chitosan–MWCNTs, and (F) curcumin–MWCNTs. Cell viability of fibroblast, PANC-1, A549, and HCT116 cell lines was assessed after exposure to increasing concentrations (12.5– 400 µg/mL) of each formulation using the MTT assay. Data are presented as mean ± SEM (n = 3).

Dose-responsive cytotoxicity curves (Fig. 2) demonstrated that low oxidized-MWCNT concentrations increased cancer cell viability while minimally affecting fibroblasts, confirming safety.

At lower concentrations, low molecular weight chitosan and chitosan-MWCNTs exhibited minimal cytotoxicity, consistent with their known biocompatibility and dose-dependent effects. Importantly, these observations do not affect the cytotoxic activity of curcumin-loaded nanoparticles, which demonstrated significant, dose-dependent reduction in cancer cell viability [11]. The study shows selective cytotoxicity toward cancer cell lines, with lower toxicity to fibroblasts, indicating preliminary biocompatibility in vitro.

Curcumin-chitosan-MWCNTs displayed selective cytotoxicity toward cancer cells, particularly PANC-1, when comparing with fibroblasts [9]. Previous studies have suggested that CNT-based systems may respond to tumor-associated microenvironmental conditions, which could potentially influence release behavior [51]. However, stimulus-responsive release was not investigated in the present study.

Enhanced cytotoxicity of curcumin-chitosan-MWCNT against PANC-1 cells may be associated with several potential mechanisms that have been reported for similar nanocarrier systems. One plausible explanation is the modulation of apoptosis-related signaling pathways, as Kuzminska, et al., who reported a change in cell apoptosis-related proteins levels—an increase in the cleaved PARP protein and a decrease in procaspase 3 and Bcl-2. At the same time, no changes in Bax expression were noticed after treating PANC-1 and BXPC-3, pancreatic cancer cell lines with curcumin derivative [52].

Another expected explanation to the observed reduction in cancer cell viability in the present study may be the association between curcumin derivatives and higher levels of Reactive oxygen species (ROS) formation, mitochondrial dysfunction, increased level of endoplasmic reticulum stress related proteins. Moreover, ROS scavenger eliminates the drop in Bcl-2 levels and cell apoptosis, indicating that ROS generation was a significant factor in cell death. The residual apoptotic impact was associated with inhibition of P-STAT3, a protein linked with apoptosis resistance in cancer cells [53]**.**

However, it is important to emphasize that these mechanistic interpretations are inferred from previously published studies and were not directly investigated in the current work. Therefore, they should be considered as plausible, hypothesis-driven explanations rather than experimentally validated mechanisms. Further studies, including quantitative cellular uptake analysis, ROS measurement, and apoptosis pathway investigation, are required to confirm these proposed mechanisms.

Chitosan functionalization increases the hydrophilicity and colloidal stability of MWCNTs while imparting a positive surface charge that may enhance interactions with the negatively charged plasma membrane, as suggested in previous studies [54]. Such surface modifications have been associated in the literature with improved dispersion and altered nano–bio interactions; however, cellular uptake and intracellular delivery were not directly evaluated in the present study. Therefore, any potential contribution of these properties to the observed cytotoxic effects remains hypothetical. Previous studies have also suggested that nanocarrier systems may support gradual release of curcumin under physiologically relevant conditions, which could potentially improve its stability and bioavailability [55]. In addition, curcumin has been reported to induce oxidative stress–related cytotoxic effects through mechanisms such as ROS generation and mitochondrial dysfunction in cancer cells [56]. However, intracellular release behavior, endosomal trafficking, curcumin accumulation, and ROS generation were not assessed in the current work. Thus, these mechanisms should be regarded only as literature-based possibilities rather than demonstrated outcomes of this study.

This aligns with Sobh et al., 2019, who reported enhanced anticancer activity of curcumin formulations with chitosan or chitosan-MWCNTs while maintaining selectivity toward normal cell lines such as RPE-1. In addition, physicochemical properties of CNT-based systems, including size, diameter, concentration, and surface functionalization, have been reported to influence their biological interactions and dispersion behavior [51]. In our previous characterization study, Rabba’a et al., 2024 [5] reported SEM micrographs showing curvy MWCNTs with variable size distributions and some degree of aggregation, likely due to intermolecular forces between nanotubes [57]. Nevertheless, no direct conclusions regarding cellular internalization mechanisms or intracellular trafficking can be drawn from the present results. Previous studies have described several possible interaction pathways between CNT-based nanomaterials and cells, including adsorption at the cell surface and various endocytic processes [51]. However, such mechanisms were not investigated in the current study, and no direct conclusions regarding cellular internalization, intracellular trafficking, or release behavior can be drawn from the present results.

Curcumin, curcumin- MWCNT, and curcumin- chitosan- MWCNTs showed dose- dependent effects on cell viability, with curcumin- chitosan- MWCNTs exhibiting greater cytotoxicity compared to free curcumin and curcumin-MWCNT. This enhanced effect may be attributed to improved dispersion, surface properties, and interactions with the cellular environment [58], as suggested in previous studies, although cellular uptake and intracellular accumulation were not directly evaluated in the present work.

Interestingly, at higher concentrations, free curcumin appeared to reduce cancer cell viability more effectively than the nanoparticle formulations. This effect may be related to the immediate availability of free curcumin in the culture medium, which is fully accessible to cells, resulting in a rapid cytotoxic response. In contrast, the reduced short-term cytotoxicity observed with curcumin-loaded nanocomposites may reflect differences in formulation characteristics, dispersion behavior, or curcumin association with the carrier matrix. Previous studies have suggested that nanocarrier systems can support more gradual release profiles which is advantageous for therapeutic applications [59]; however, release kinetics were not directly evaluated in the present study. Therefore, any interpretation related to sustained release or delivery performance should be considered hypothetical.

All treatments exhibited selective cytotoxicity, with PANC-1, A549, and HCT116 cancer cells showing lower viability than fibroblasts. Maximum cytotoxicity for fibroblasts was 60.12% with 200 µg/mL oxidized-MWCNT, as MWCNTs can induce apoptosis and membrane damage [60] while chitosan-bound MWCNTs showed 77.1% cytotoxicity, exceeding MWCNTs or chitosan alone [54]. These results highlight the balance between efficacy and biocompatibility achieved by the designed nanocomposites.

Curcumin demonstrated over 80% cytotoxicity at 400–50 µg/mL, and in combination with MWCNT, was safe at 400 µg/mL, with 10–29% toxicity between 12.5 and 200 µg/mL, indicating MWCNT reduces curcumin's toxicity. Several studies have shown that curcumin's cytotoxic effects increase with concentration, risking damage and apoptosis in malignant and normal cells, but was generally safe at lower doses [55]. This toxicity stems from reactive oxygen species (ROS), mitochondrial disruption, and caspase-mediated apoptosis [56]. High concentrations may trigger stress, affecting pro- and anti-apoptotic pathways [61]. Despite therapeutic potential, curcumin' s cytotoxicity raises safety concerns with long-term high doses [62].

In A549 cells, the highest cytotoxicity occurred at 400 µg/mL across various treatments, consistent with Ursini et al., which linked high cytotoxicity to membrane damage and increased IL-6 and IL-8. There's a correlation between cellular MWCNT uptake and negatively charged surface groups (-OH and -COOH), enhancing transport [60]. For PANC-1, cytotoxicity at 400 µg/mL reached 87.22%, 84.3%, 89.4%, 88.3%, 86.2%, and 77.19% for various treatments, while the lowest was 25.5%, 1.08%, and 0% for lower doses of oxidized-MWCNT, chitosan, and curcumin [63].

In 2020, Badea et al. tested Quercetine-Pemetrexed-MWCNT (MWCNT-Pm-Q) on pancreatic (PANC-1) and breast (MDA-MB-231) cancer cell lines, finding a 40% decrease in PANC-1 cell viability at 25 μg/mL, compared to a 14% decrease in MDA-MB-231. Co-delivery with MWCNT enhanced oxidative stress and proliferation inhibition more effectively than separate drug administration. This also confirms that MWCNTs are considered an excellent vehicle used to target drug delivery to specific cancer locations [63].

Curcumin combats cancer by regulating epigenetic modifications. In colorectal cancer cells, treatment with 7.5–10 μM curcumin for 6 days causes demethylation of specific CpG loci [64]. It inhibits DNA methyltransferase to activate tumor suppressor genes but may also promote hypermethylation of oncogene promoters [65] where mTOR is the abbreviation of mammalian target of rapamycin promoter which is supposed to be needed for several biological activities including cell proliferation [63], [66].

Although the curcumin–chitosan–MWCNT formulation exhibited enhanced anticancer activity, the study did not directly assess intracellular curcumin uptake. Therefore, further research using quantitative analytical techniques is needed to confirm delivery efficiency and clarify cellular uptake mechanisms. Moreover, while the system showed encouraging *in vitro* anticancer results, additional *in vivo* studies on toxicity, bio-distribution, clearance, and long-term safety are essential to establish its clinical safety, especially considering existing safety issues associated with some MWCNT types.

To further contextualize the cytotoxic performance of the developed nanocomposite, the IC₅₀ values obtained in this study (PANC-1: 67 µg/mL; A549: 148.6 µg/mL; HCT116: 71.4 µg/mL; fibroblasts: 227.6 µg/mL) were compared with those reported for similar curcumin-based nanocarrier systems. For instance, Zainol Abidin et al., reported that curcumin loaded into chitosan-gold nanoparticles (CCG-NP) exhibited an IC_50_ of approximately 58% for the NIH 3T3 cell line (fibroblast cell line derived from embryonic mouse tissue) [67]. Similarly, Ali et al. demonstrated that CUR-Liposomes showed an IC₅₀ of around 87.43 μg/mL for MCF-7 treated with CUR-Liposomes [68]. In another study, Almutairi, et al. reported IC_50_ values of 150 µM for Curcumin-loaded chitosan nanoparticles treated A549 (lung cancer) [69]. Also in 2023, Ilhan, obtained IC_50_ value of 267 µg/mL for HepG2 (hepatocellular carcinoma) treated with Curcumin carbon-dot chitosan nanoparticles [70]. In addition, *in vitro* treated HeLa (cervical cancer) cells with Curcumin-loaded chitosan nanoparticles showed 24 µM IC_50_ value [71], whereas, SiHa (cervical cancer) cells *in vitro* treated with Ionic gelation curcumin-loaded chitosan nanoparticles showed IC_50_ value of 4 mg/mL [72]. In another similar *in vitro* study used Curcumin-loaded chitosan-grafted halloysite nanotubes the IC_50_ value for bladder cancer cell line EJ was 5.3 µM [73]. Here it is important to emphasize that direct comparison of IC₅₀ values across studies should be interpreted with caution due to variations in experimental conditions, including differences in cell lines, incubation times, and assay protocols. Therefore, the present comparison is intended to provide a general benchmark rather than a definitive evaluation of relative efficacy.

Moreover, while the system showed promising *in vitro* anticancer results, additional *in vivo* studies on toxicity, bio-distribution, clearance, and long-term safety are essential, given existing safety concerns associated with some MWCNT types.

Also it is important to note that cellular uptake and intracellular trafficking of the developed nanocomposite were not directly investigated in this study. Therefore, any proposed enhancement in intracellular delivery or uptake is inferred from previously reported studies on similar nanocarrier systems and should be considered as a plausible hypothesis rather than an experimentally validated conclusion. Future studies employing quantitative uptake assays (e.g., fluorescence imaging or flow cytometry) are required to confirm these assumptions.

## 5 Conclusions

Multi-walled carbon nanotubes (MWCNTs) are widely investigated in nanomedicine; however, their limited aqueous dispersibility necessitates surface functionalization prior to biomedical application. In the present study, chitosan functionalization was employed to improve the physicochemical properties and dispersion behavior of MWCNTs. The successful association of curcumin with both pristine and chitosan-functionalized MWCNTs was supported by zeta potential and XRD analyses.

The in vitro cytotoxicity of curcumin–chitosan–MWCNTs and curcumin–MWCNTs was evaluated against PANC-1, HCT116, A549, and fibroblast cell lines, where both formulations exhibited enhanced cytotoxic effects compared to free curcumin, with selective activity toward cancer cells. These findings support the potential of the developed nanocomposite formulations as selective in vitro anticancer systems.

However, it is important to emphasize that the present study did not include direct evaluation of cellular uptake, intracellular localization, or drug release behavior within the tested cell lines. Therefore, the role of chitosan-functionalized MWCNTs as an effective drug delivery system cannot be conclusively established based on the current data and should be considered a potential application requiring further investigation.

Further research should focus on elucidating the mechanisms underlying the observed cytotoxicity and selectivity of the curcumin–chitosan–MWCNT formulation. Future studies should include quantitative assessments of cellular uptake, intracellular trafficking, and release kinetics using validated analytical techniques such as LC–MS/HPLC or fluorescence-based assays to better evaluate its potential as a nanocarrier system.

## CRediT authorship contribution statement

**Manar M. Rabba’a:** Writing – review & editing, Writing – original draft, Methodology, Investigation, Formal analysis, Data curation. **Bashaer Abu-Irmaileh:** Writing – review & editing, Methodology, Formal analysis. **Saida Abu Mallouh:** Writing – review & editing, Methodology, Formal analysis. **Aya Khalaf:** Writing – review & editing, Investigation. **Abu-Zurayk Rund:** Writing – review & editing, Supervision, Project administration, Methodology, Conceptualization. **Yaser K. Bustanji:** Writing – review & editing, Supervision, Methodology, Conceptualization.

## Declaration of Competing Interest

The authors declare that they have no known competing financial interests or personal relationships that could have appeared to influence the work reported in this paper.

## Data Availability

Data will be made available on request.
